# Supplementary material for: Integrated computational and experimental immunoengineering of adeno-associated virus capsid T cell epitopes in mice
Source: Nat Commun. 2026 Mar 3;17:3387. doi: 10.1038/s41467-026-69917-9 (PMC13065821; doi:10.1038/s41467-026-69917-9)

Supplementary Table 1. In silico analysis of identified epitope

Peptide #	Sequence	Percentile rank	
		H2-IAd	H2-IEd
102	NNWGFRPKRLNFKLF	86	1.2
	NWGFRPKRLNFKLFN	84	1.6
	WGFRPKRLNFKLFNI	82	2.7
103	GFRPKRLNFKLFNIQ	77	13

Binding affinity prediction using NetMHCII2.3 method, which was the most up-to-date version available at the time of analysis

Supplementary Table 2. Comparison of Predicted MHCII Binding Affinity for Wild-Type and Mutant AAV9 Epitopes

peptide		consensus	NetMHCII1.1 (SMM-align)	NetMHCII2.3 (NN-align 2.3)	NetMHCIIpan4.3 EL	NetMHCIIpan4.2 EL	NetMHCIIpan4.1 EL
**NWGFRPKRLNFKLFN**	**WT**	**6.8**	**12**	**1.6**	**0.83**	**0.82**	**0.7**
NWGFRPK**H**LNFKLFN	R312H	16	19	13	0.52	0.55	0.56
NWGFRPK**Q**LNFKLFN	R312Q	22.5	33	12	0.83	0.75	0.77

Supplementary figure 1

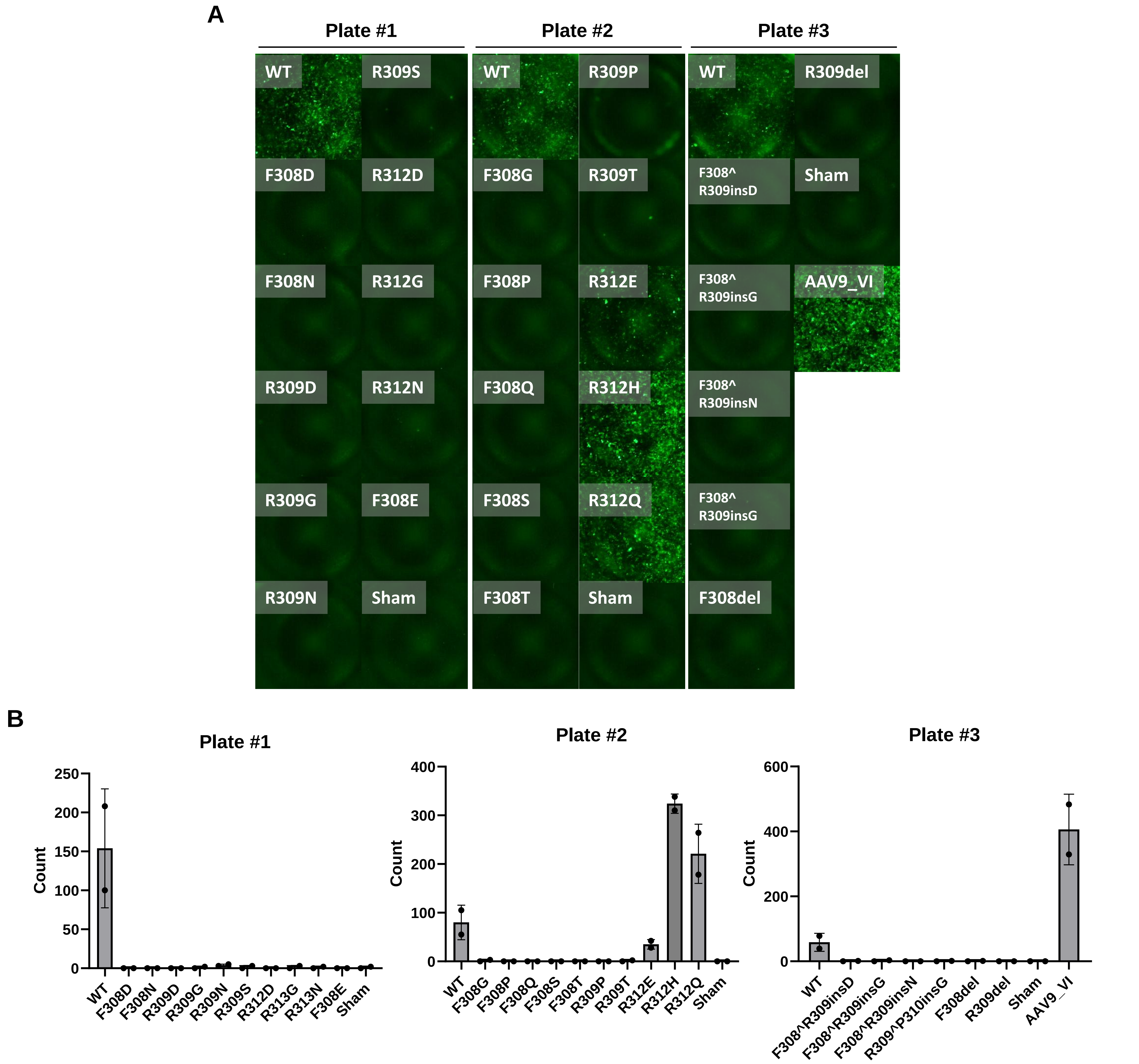

Supplementary Figure 1. Analysis of GFP-expressing HeLa cells using ELISpot reader. A total of 26 mutated AAV variants carrying the GFP transgene were isolated using the method described in Figure 4. Viral production cells (VPCs) were transfected in three 12-well plates, each including wild-type (WT) AAV9 and a sham control. The precipitated AAVs were directly applied to HeLa cells and incubated for 3 days. (A) GFP-expressing HeLa cells were imaged using an ELISpot reader. (B) GPF-expressing cells were then counted using ImmunoSpot software. Each sample was prepared in duplicate, with one replicate in each of two separate plates. This experiment was repeated three times independently with similar results. Values are presented as mean ± SD.

Supplementary figure 2

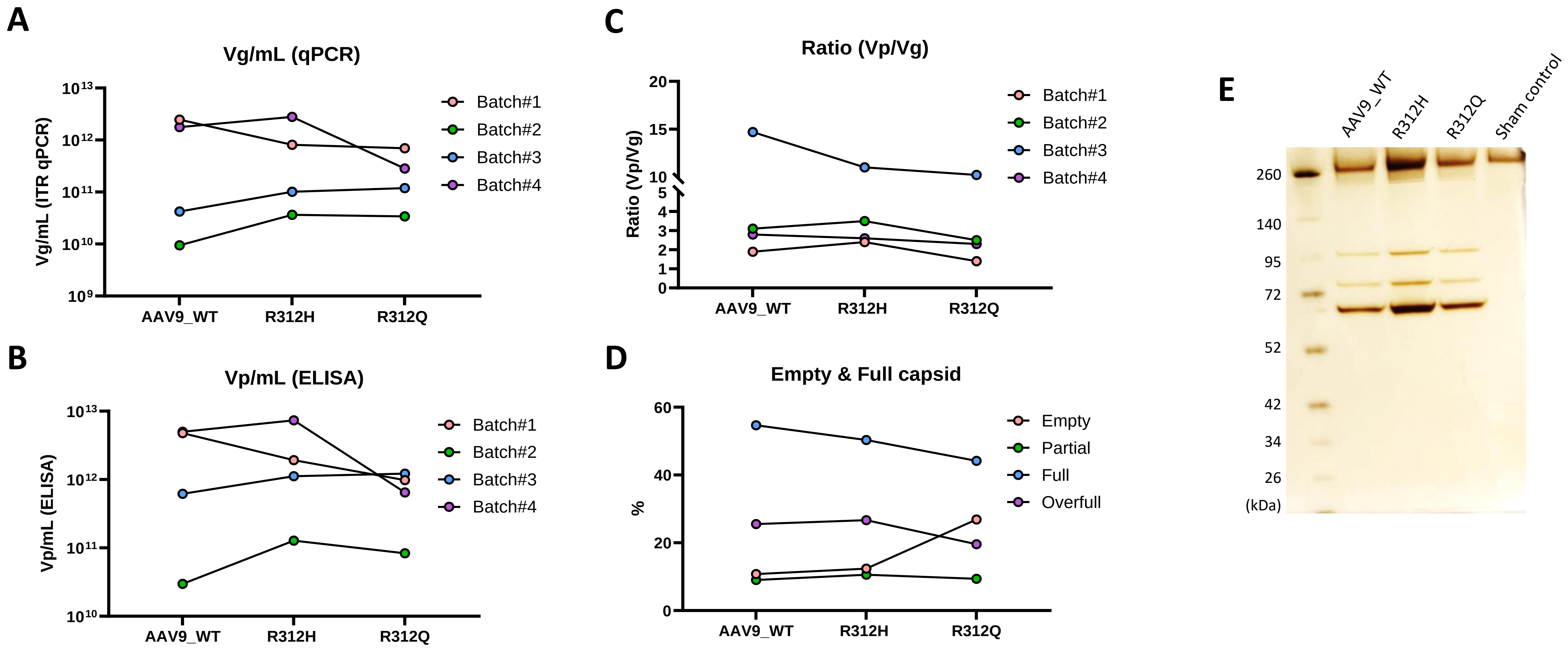

Supplementary Figure 2. Mutations in AAV9 capsid had no effect on the production yield or size of the vector. (A–C) Viral production metrics were assessed across four independent manufacturing batches (Batch #1–4) produced by triple transfection of HEK293 cells. Data show (A) viral genome concentration (Vg/mL) determined by ITR-targeting qPCR, (B) total viral particles (Vp/mL) measured by AAV9 ELISA, and (C) the ratio of viral particles to viral genomes (Vp/Vg). Individual data points for each batch are represented by colored circles and connected by lines to show trends across variants. (D) Physical occupancy of AAV capsids (Empty, Partial, Full, and Overfull) in a representative batch, quantified by charge-detection mass spectrometry (CDMS) and expressed as a percentage (%) of the total population. (E) Representative silver-stained SDS-PAGE gel showing the viral proteins (VP1, VP2, and VP3) of WT and mutated AAVs to assess structural integrity. Molecular weight markers are indicated in kilodaltons (kDa) on the left. Data in (E) are representative of two independent experiments with similar results.

Supplementary figure 3

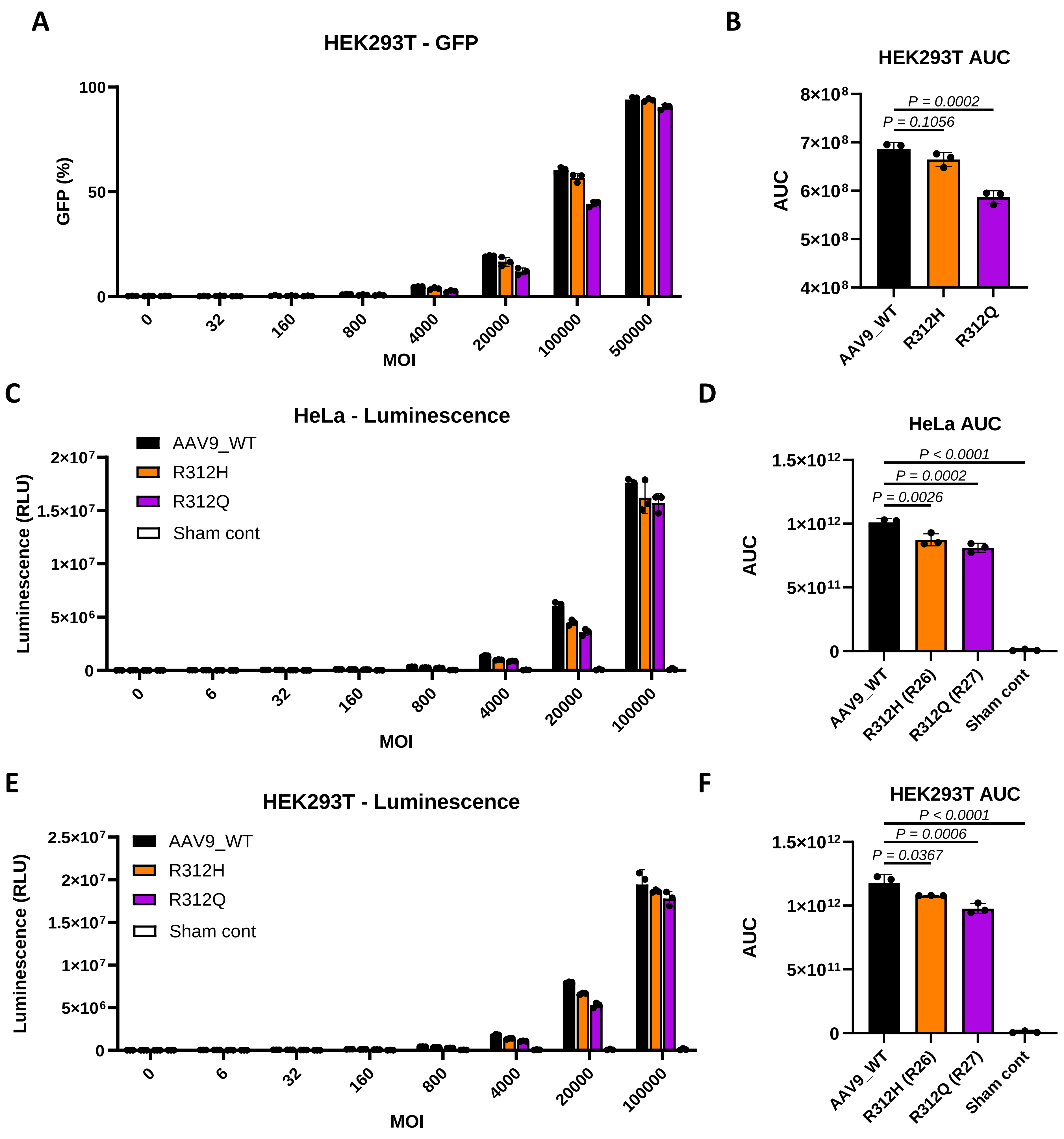

Supplementary Figure 3. Comparison of the transduction efficiency of WT AAV9 and mutated AAVs with other cell line or other transgene (NanoLuc) (A, B) HEK293T cells were transduced with the AAV9, R312H, and R312Q vectors at an indicated MOI. The percentage of GFP-positive cells was determined by flow cytometry. HeLa cells (C, D) and HEK293T cells (E, F) were transduced with the AAV9, R312H, and R312Q vectors that express NanoLuc at an indicated MOI. Next day, bioluminescence intensities of the cells were determined by luminometer. (B, D, F) Data are shown as a representative result from three independent experiments with similar results. For each experiment, n = 3 independent biological replicates were used. Four-parameter curve fit was calculated for each vector and area under the curve (AUC) were calculated. Each bar shows the mean ± SD. P values were determined by one way ANOVA with Tukey's multiple comparisons test. Source data are provided as a Source Data file. MOI, multiplicity of infection; AUC, area under the curve; Sham, sham control.

Supplementary figure 4

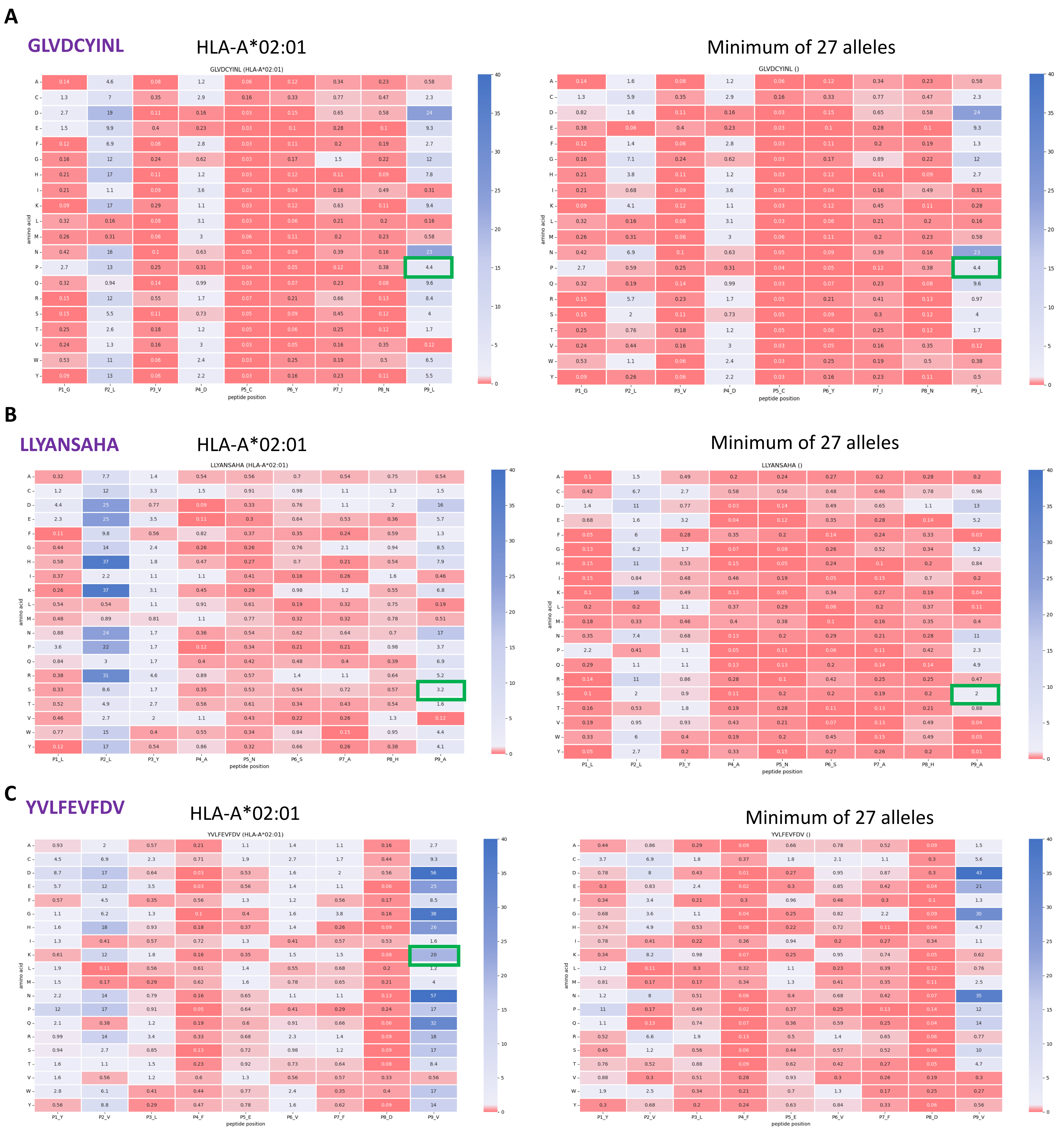

Supplementary Figure 4. Validation of EMMP using three reported HLA-A02:01-restricted CD8+ T cell epitopes in hexon protein
We applied it to three reported HLA-A02:01-restricted CD8+ T cell epitopes in hexon protein (A: GLVDCYINL, B: LLYANSAHA, C: YVLFEVFDV). EMMP analysis successfully identified substitutions predicted to reduce MHC binding (colored in blue), with results for each epitope shown below in sequential order for both HLA-A02:01-specific binding (left panels) and minimum binding values across all 27 representative alleles (right panels). Notably, several computationally predicted mutations corresponded to empirically validated modifications described in the granted patent (L520P, A900S, V925K, highlighted in green boxes)

CDMS data used to make supplementary figure 2D

AAV9_WT 01

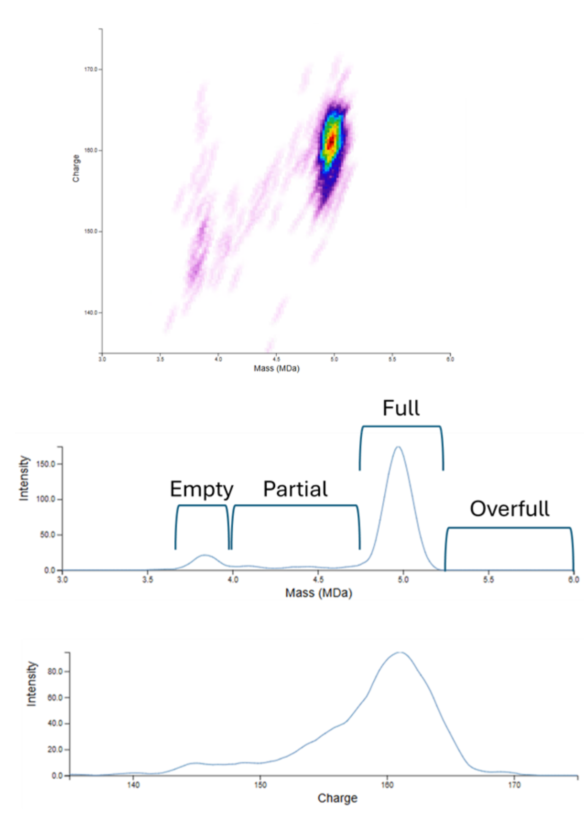

AAV9_WT 02

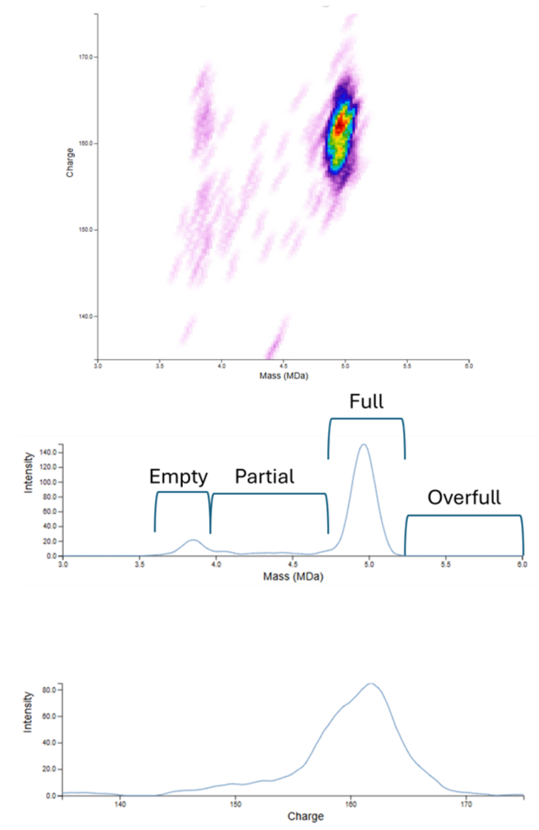

AAV9_WT 03

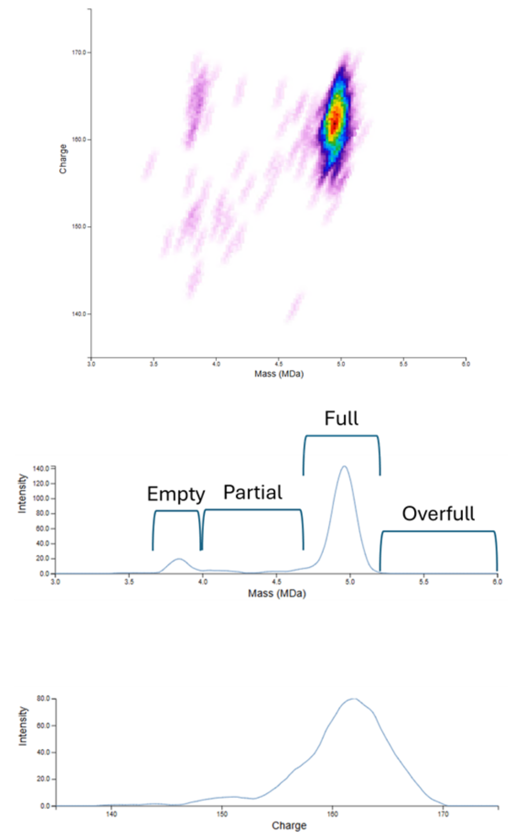

R312H 01

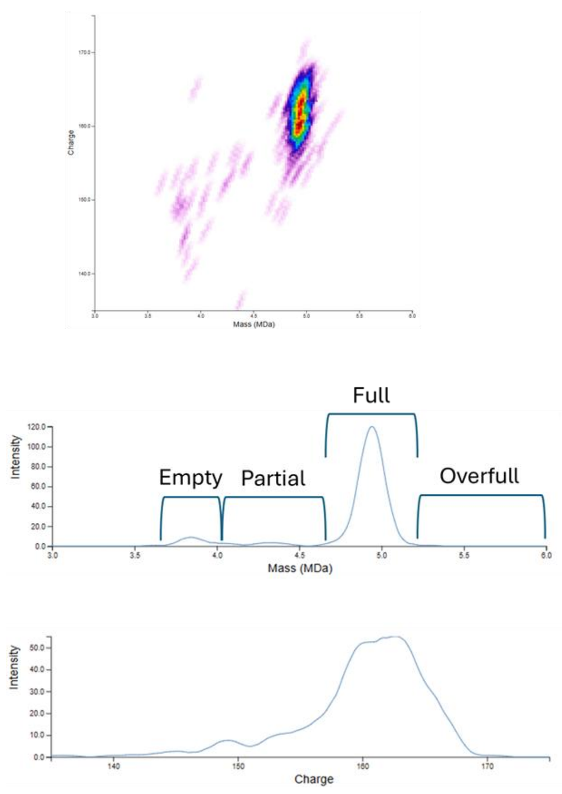

R312H 02

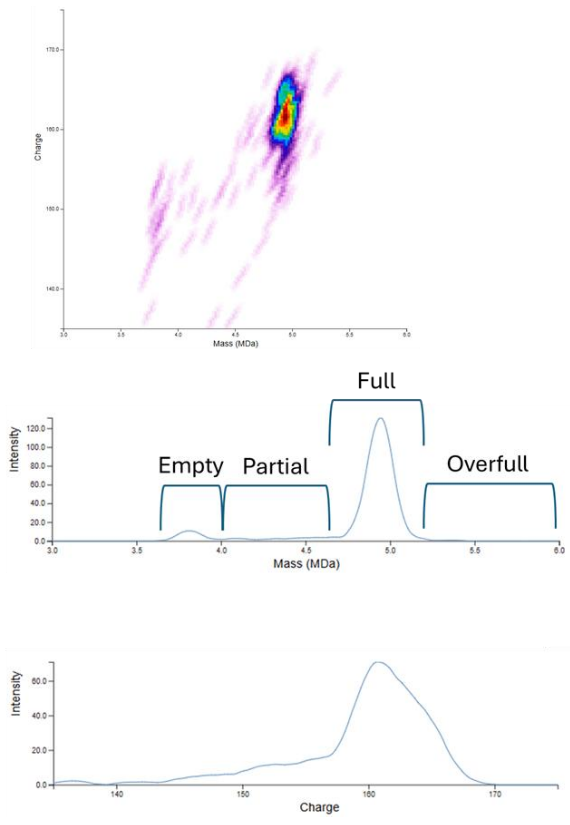

R312H 03

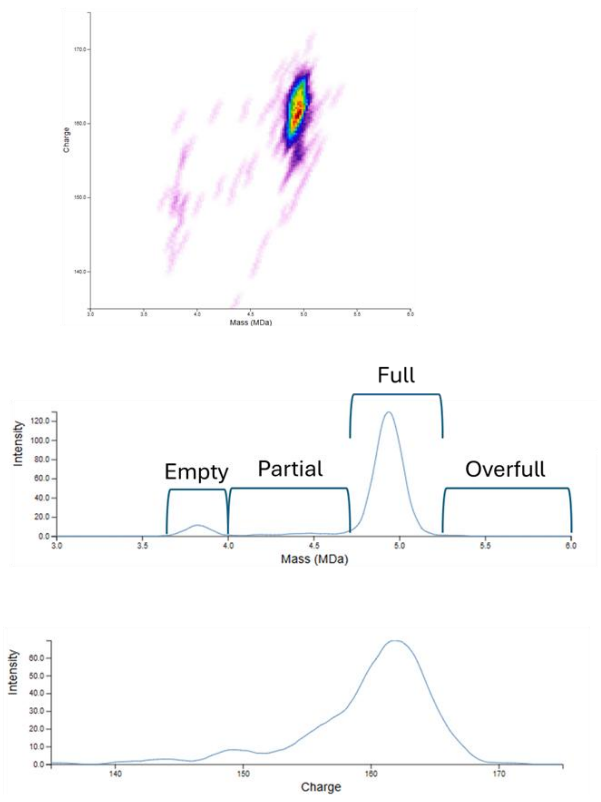

R312Q 01

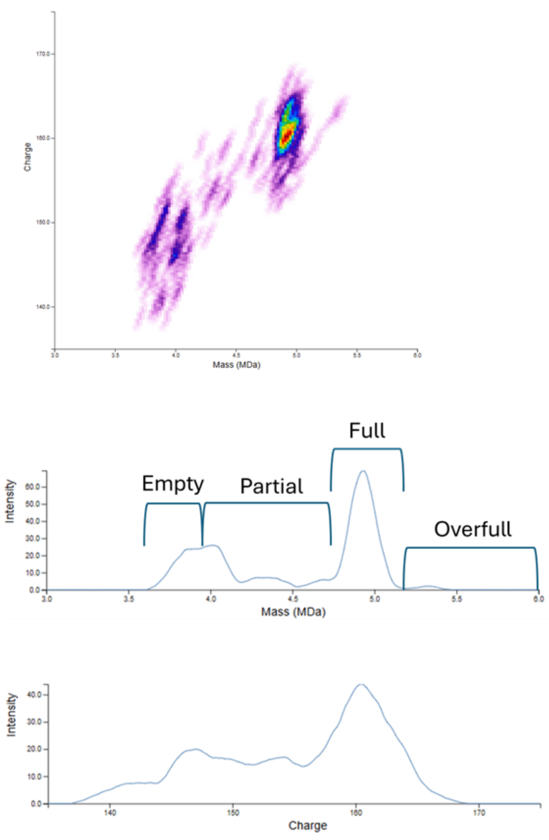

R312Q 02

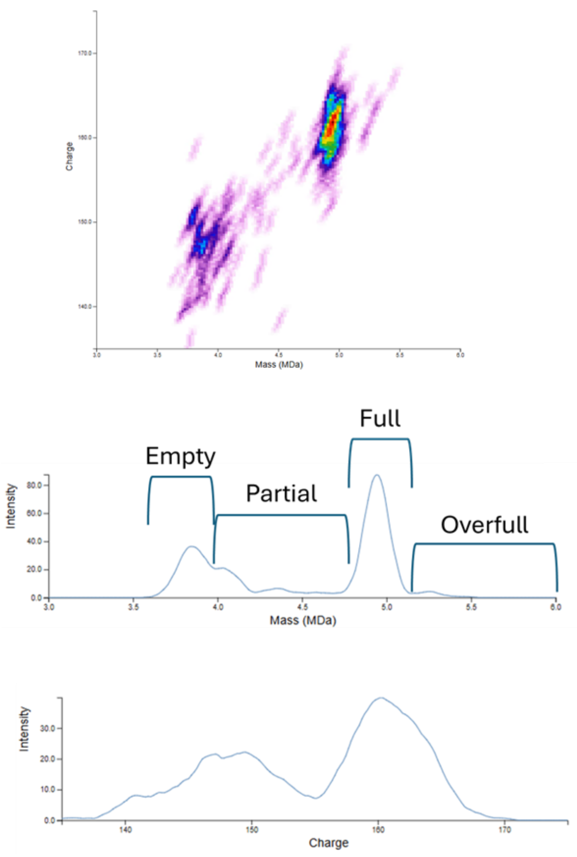

R312Q 03

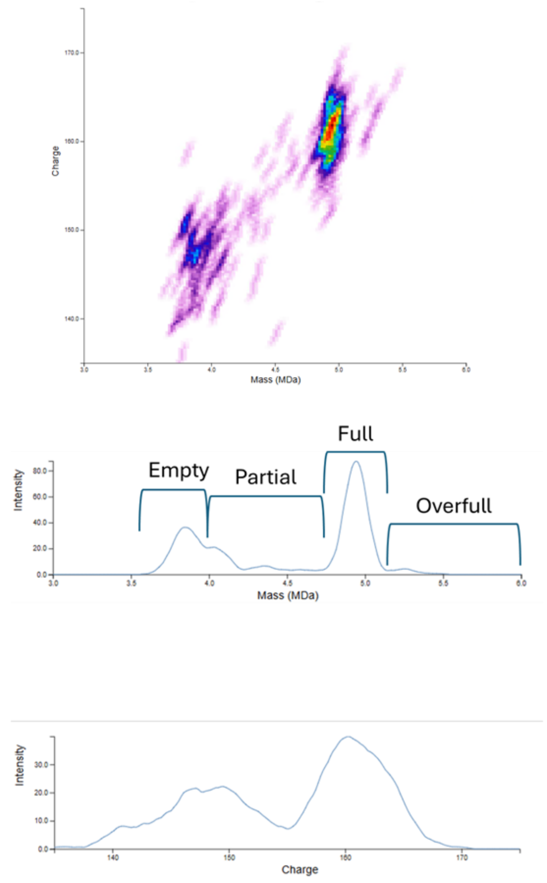

Uncropped scan of gels in supplementary figure 2E

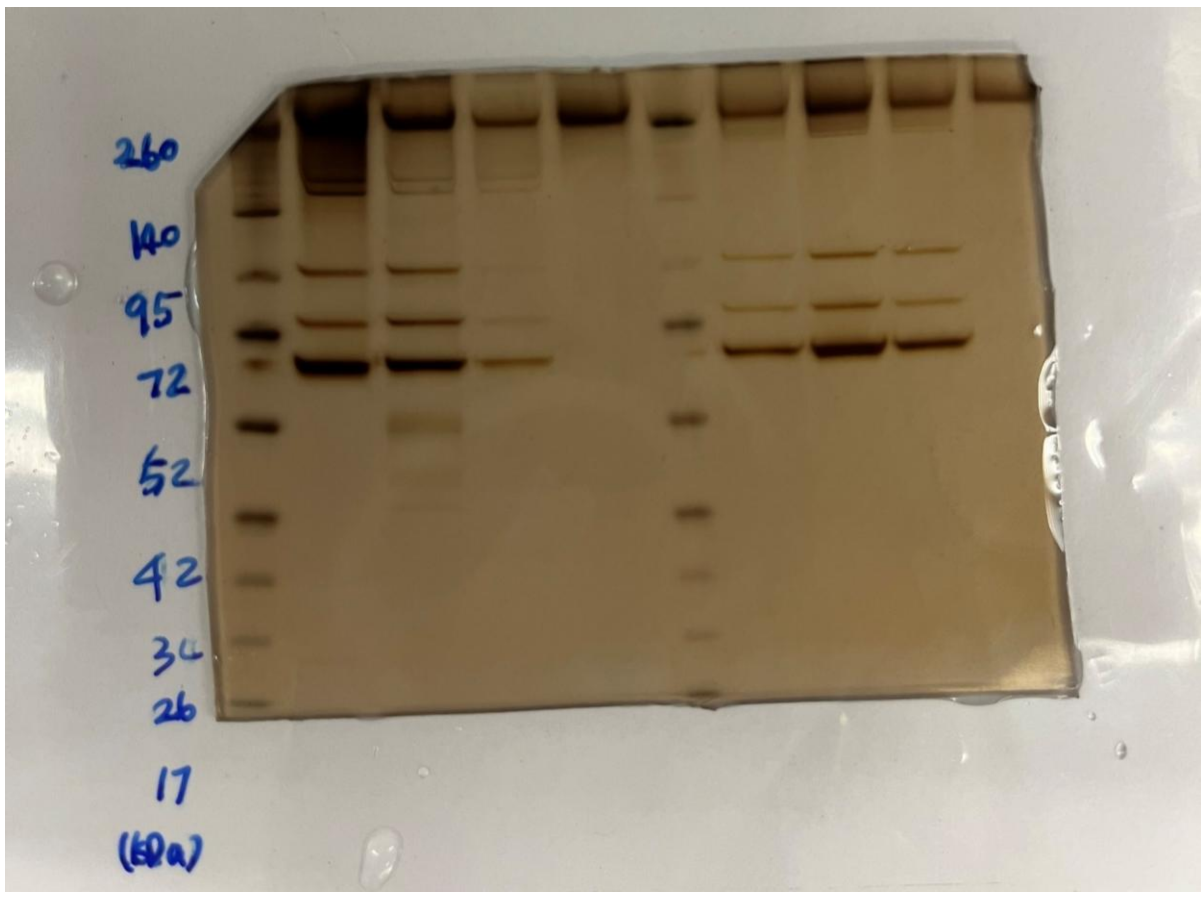

Supplement: Supplementary file 1 — Supplementary Information [file 41467_2026_69917_MOESM1_ESM.pdf]
