# Supplementary material for: Integrated computational and experimental immunoengineering of adeno-associated virus capsid T cell epitopes in mice
Source: Nat Commun. 2026 Mar 3;17:3387. doi: 10.1038/s41467-026-69917-9 (PMC13065821; doi:10.1038/s41467-026-69917-9)
Supplement: Supplementary file 2 — Reporting Summary [file 41467_2026_69917_MOESM2_ESM.pdf]

nature portfolio

Corresponding author(s): Ronit Mazor

Last updated by author(s): Jan 14, 2026

Reporting Summary

Nature Portfolio wishes to improve the reproducibility of the work that we publish. This form provides structure for consistency and transparency in reporting. For further information on Nature Portfolio policies, see our Editorial Policies and the Editorial Policy Checklist.

Statistics

For all statistical analyses, confirm that the following items are present in the figure legend, table legend, main text, or Methods section.

n/a	Confirmed	
☐	☒	The exact sample size (*n*) for each experimental group/condition, given as a discrete number and unit of measurement
☐	☒	A statement on whether measurements were taken from distinct samples or whether the same sample was measured repeatedly
☐	☒	The statistical test(s) used AND whether they are one- or two-sided<br>*Only common tests should be described solely by name; describe more complex techniques in the Methods section.*
☒	☐	A description of all covariates tested
☒	☐	A description of any assumptions or corrections, such as tests of normality and adjustment for multiple comparisons
☐	☒	A full description of the statistical parameters including central tendency (e.g. means) or other basic estimates (e.g. regression coefficient) AND variation (e.g. standard deviation) or associated estimates of uncertainty (e.g. confidence intervals)
☐	☒	For null hypothesis testing, the test statistic (e.g. *F*, *t*, *r*) with confidence intervals, effect sizes, degrees of freedom and *P* value noted<br>*Give P values as exact values whenever suitable.*
☒	☐	For Bayesian analysis, information on the choice of priors and Markov chain Monte Carlo settings
☒	☐	For hierarchical and complex designs, identification of the appropriate level for tests and full reporting of outcomes
☒	☐	Estimates of effect sizes (e.g. Cohen's *d*, Pearson's *r*), indicating how they were calculated

Our web collection on statistics for biologists contains articles on many of the points above.

Software and code

Policy information about availability of computer code

Data collection	Cytek Aurora (Cytek Biosciences, Fremont, CA), www.iedb.org, Immunospot 7.0 (Cellular Technology Limited, Cleveland, OH), IVIS spectrum imager (PerkinElmer, Waltham, MA)
Data analysis	GraphPad Prism 10.2 (GraphPad Software Inc., San Diego, CA, USA), Flowjo v10.1.0 (Treestar, Ashland, OR), IVIS spectrum imager (PerkinElmer, Waltham, MA)

For manuscripts utilizing custom algorithms or software that are central to the research but not yet described in published literature, software must be made available to editors and reviewers. We strongly encourage code deposition in a community repository (e.g. GitHub). See the Nature Portfolio guidelines for submitting code & software for further information.

Data

Policy information about availability of data

All manuscripts must include a data availability statement. This statement should provide the following information, where applicable:

- Accession codes, unique identifiers, or web links for publicly available datasets
- A description of any restrictions on data availability
- For clinical datasets or third party data, please ensure that the statement adheres to our policy

A reporting summary for this article is available as Supplementary Information file. The data supporting the findings of this study are available within the article and

its Supplementary Figs. Any additional information required to reanalyze the data reported in this paper is available from the lead contact upon request. Source data are provided with this paper.

Research involving human participants, their data, or biological material

Policy information about studies with human participants or human data. See also policy information about sex, gender (identity/presentation), and sexual orientation and race, ethnicity and racism.

Reporting on sex and gender	N/A
Reporting on race, ethnicity, or other socially relevant groupings	N/A
Population characteristics	N/A
Recruitment	N/A
Ethics oversight	N/A

Note that full information on the approval of the study protocol must also be provided in the manuscript.

Field-specific reporting

Please select the one below that is the best fit for your research. If you are not sure, read the appropriate sections before making your selection.

☒ Life sciences ☐ Behavioural & social sciences ☐ Ecological, evolutionary & environmental sciences

For a reference copy of the document with all sections, see nature.com/documents/nr-reporting-summary-flat.pdf

Life sciences study design

All studies must disclose on these points even when the disclosure is negative.

Sample size	No statistical method was used to predetermine sample size, which was chosen based on prior experience and standard practice in the field to ensure adequate power for comparison.
Data exclusions	In Figure 7A and 7B, data from certain wells were excluded from the final analysis because of technical issues during the plate washing process, where a clogged nozzle prevented proper washing.
Replication	All experiments were repeated at least twice, with similar results.
Randomization	Mice were assigned randomly to the experimental groups.
Blinding	The investigators were blinded to group allocation during data collection and outcome assessment to minimize bias. Samples were coded by a separate researcher so that the investigators performing the assays were unaware of the specific group identities until the final analysis was completed.

Reporting for specific materials, systems and methods

We require information from authors about some types of materials, experimental systems and methods used in many studies. Here, indicate whether each material, system or method listed is relevant to your study. If you are not sure if a list item applies to your research, read the appropriate section before selecting a response.

Materials & experimental systems

n/a	Involved in the study	
☐	☒	Antibodies
☐	☒	Eukaryotic cell lines
☒	☐	Palaeontology and archaeology
☐	☒	Animals and other organisms
☒	☐	Clinical data
☒	☐	Dual use research of concern
☒	☐	Plants

Methods

n/a	Involved in the study	
☒	☐	ChIP-seq
☐	☒	Flow cytometry
☒	☐	MRI-based neuroimaging

Antibodies

Antibodies used	CD3 (1:1000, clone 500A2, BioLegend, cat. 560771, RRID: AB_2564590), CD4 (1:1000, clone RM4-5, BioLegend, cat. 100528, RRID: AB_312729), CD8 (1:1000, clone 53-6.7, BioLegend, cat. 100706, RRID: AB_312745), CD69 (1:1000, clone H1.2F3, BioLegend, cat. 104512, RRID: AB_493564), IFN-γ (1:500, clone XMG1.2, BioLegend, Cat. 505808, RRID: AB_315402) and TNF-α (1:500, clone MP6-XT22, BioLegend, Cat. 506328, RRID: AB_2562902), and anti-mouse IgG antibodies conjugated to HRP (Jackson Laboratory, cat. 115-035-166, RRID:AB_2338511)
Validation	Antibody validation was provided by manufacture's website. Antibodies were validated by testing different antibody clones to optimize signal-to-noise ratio and to ensure accurate identification of cellular markers.

Eukaryotic cell lines

Policy information about cell lines and Sex and Gender in Research

Cell line source(s)	HeLa (CCL-2) and HEK293T (CRL-3216) cell lines were obtained from ATCC. Viral Production Cells 2.0 (A49784) was obtained from Thermo Fisher Scientifc
Authentication	The cell lines were authenticated by the suppliers. No additional authentication was performed by the authors.
Mycoplasma contamination	All cell lines tested negative for mycoplasma contamination.
Commonly misidentified lines (See ICLAC register)	No commonly misidentified cell lines were used.

Animals and other research organisms

Policy information about studies involving animals; ARRIVE guidelines recommended for reporting animal research, and Sex and Gender in Research

Laboratory animals	6–8-week-old BALB/c mice were purchased from The Jackson Laboratory
Wild animals	The study did not involve wild animals.
Reporting on sex	Female mice were used to ensure experimental consistency and to minimize physiological variability
Field-collected samples	The study did not involve samples collected from the field.
Ethics oversight	Animal protocols were approved by the FDA White Oak Consolidated Animal Care and Use Committee (protocols 2023–18)

Note that full information on the approval of the study protocol must also be provided in the manuscript.

Plants

Seed stocks	*Report on the source of all seed stocks or other plant material used. If applicable, state the seed stock centre and catalogue number. If plant specimens were collected from the field, describe the collection location, date and sampling procedures.*
Novel plant genotypes	*Describe the methods by which all novel plant genotypes were produced. This includes those generated by transgenic approaches, gene editing, chemical/radiation-based mutagenesis and hybridization. For transgenic lines, describe the transformation method, the number of independent lines analyzed and the generation upon which experiments were performed. For gene-edited lines, describe the editor used, the endogenous sequence targeted for editing, the targeting guide RNA sequence (if applicable) and how the editor was applied.*
Authentication	*Describe any authentication procedures for each seed stock used or novel genotype generated. Describe any experiments used to assess the effect of a mutation and, where applicable, how potential secondary effects (e.g. second site T-DNA insertions, mosiacism, off-target gene editing) were examined.*

Flow Cytometry

Plots

Confirm that:

- [ ] The axis labels state the marker and fluorochrome used (e.g. CD4-FITC).
- [ ] The axis scales are clearly visible. Include numbers along axes only for bottom left plot of group (a 'group' is an analysis of identical markers).
- [ ] All plots are contour plots with outliers or pseudocolor plots.
- [ ] A numerical value for number of cells or percentage (with statistics) is provided.

Methodology

Sample preparation	Sample preparation is described in Methods.
Instrument	Cytek Aurora
Software	FlowJo were used for data analysis.
Cell population abundance	Data on the abundance of relevant cell populations are provided in the paper.
Gating strategy	It's not provided in the manuscript.

☐ Tick this box to confirm that a figure exemplifying the gating strategy is provided in the Supplementary Information.
